# Reconstruction With Pedicled Anterolateral Thigh Flap After Wide Local Excision of Penoscrotal Extramammary Paget's Disease: A Case Report and Comprehensive Literature Review

**Published:** 2015-06-24

**Authors:** Cedric L. Hunter, Eila C. Skinner, Gordon K. Lee

**Affiliations:** ^a^Division of Plastic Surgery, Stanford University Medical Center, Palo Alto, Calif; ^b^Department of Urology, Stanford University Medical Center, Palo Alto, Calif

**Keywords:** extramammary Paget’s disease, anterolateral thigh flap, wide local excision, penoscrotal reconstruction

## Abstract

**Objective:** The clinical characteristics, management, and prognostic indicators of penoscrotal extramammary Paget's disease are not clearly defined. Surgical excision is often an effective treatment modality but results in a large wound after resection of all involved tissues. **Methods:** Reconstruction of large penoscrotal soft-tissue defects after wide local excision remains a challenge to the reconstructive surgeon. The use of the anterolateral thigh flap for penoscrotal reconstruction after resection of extramammary Paget's disease is infrequent as a reconstruction tool throughout the literature. **Results:** We discuss a case where the anterolateral thigh flap was effectively used for reconstruction of a large penoscrotal defect after wide local excision of penoscrotal extramammary Paget's disease and present a comprehensive literature review of extramammary Paget's disease key features, diagnosis, prognosis, and treatment. **Conclusions:** The anterolateral thigh flap is a useful tool for penoscrotal defect reconstruction.

In 1874, Sir James Paget described a disease process in which certain chronic conditions of the skin of the nipple and areola often preceded breast cancer. He described the appearance of an intensely red, raw surface that resembled acute diffuse eczema on the whole or greater part of the nipple and areola with an associated clear, yellowish, exudate. He noted that this chronic skin disease was frequently associated with underlying breast cancer.^1^ In 1889, Crocker^2^ described the first case of extramammary Paget's disease (EMPD) affecting the scrotum and penis. Since these initial descriptions, EMPD has been reported in multiple locations including the axilla, perineum, and groin.^3^

In general, wide local excision (WLE) is the standard treatment option and offers a possibility of cure for EMPD.^3^^-^5 However, because of delayed presentation, the defect is often too large for primary closure and other techniques such as split-thickness skin grafting or local flaps are needed for reconstruction. In this article, a case is presented discussing a more robust option for reconstruction, the anterolateral thigh (ALT) flap. At this time, only a single report is identified that mentions the use of the ALT flap for penoscrotal reconstruction after WLE of EMPD.^6^ In the literature, most studies focus on EMPD of the vulva and perineum whereas there are only small series that address penoscrotal EMPD.^3^^,^^4^^,^^4^^,^^7^ Thus, in addition to our case report, we present a review of the literature for the theory of development and progression, the relationship to internal malignancy, the treatment modalities, and the incidence of recurrence of penoscrotal EMPD, with the goal of improving our limited understanding of this rare neoplasm.[Fig F1][Fig F2]

## METHODS

The patient was a 75-year-old man of Chinese ancestry who presented with a 7-year history of right scrotal irritation and itching that acutely worsened over the past 2 years. The involved area was initially treated by his primary care physician with topical antifungal cream, but there was no improvement. He subsequently underwent a biopsy that revealed EMPD. On examination, there were no clinically evident enlarged lymph nodes. The involved area was erythematous and moist, measuring approximately 10 × 10 cm, involving the right scrotal skin and the right groin skin. Workup for internal malignancy showed negative findings.

**Figure 1 F1:**
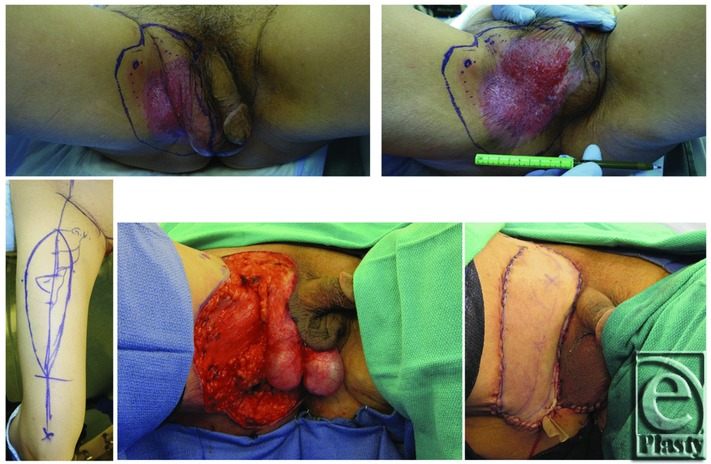
ALT flap used for reconstruction after wide local excision of EMPD. (Top, left and right) Penoscrotal EMPD before WLE with 3-cm surgical margin. (Below, left) Markings for right thigh ALT flap with marked perforators. (Bottom, center) Wound after resection of EMPD lesion full thickness down to fascia. (Bottom, right) Reconstruction of soft-tissue defect with the pedicle ALT flap after standard elevation and tunneling beneath the rectus femoris and sartorius muscles.

The patient underwent WLE of the lesion under general anesthesia. The lesion was excised full thickness down to the fascia, removing both the groin and scrotal skin with an initial 3-cm margin from the grossly involved skin. Intraoperative frozen section analysis (FSA) was used to evaluate intermittent areas at the border. All frozen section biopsies showed negative findings intraoperatively. The final wound was 240 cm^2^. The ALT flap was then raised in standard fashion from the right thigh. As is routine in our practice, while elevating the fasciocutaneous flap, perforators to the flap were identified and protected. To increase bulk and perfusion, a small cuff of the vastus lateralis muscle was harvested with the flap. The pedicle, descending branch of the lateral circumflex femoral artery, was mobilized to allow the flap to be tunneled beneath the rectus femoris and sartorius muscles and into the defect. We were careful to avoid injury to the branches of femoral nerves. The muscle was then secured proximally to limit tension on the pedicle. The remaining scrotal skin was mobilized to assist with testicular coverage. The skin and fascia of the flap were then inset into the defect over a 19-French Blake drain (Fig 1). Surgical pathology report revealed complete excision of the lesion with clear surgical margins.

## RESULTS

**Figure 2 F2:**
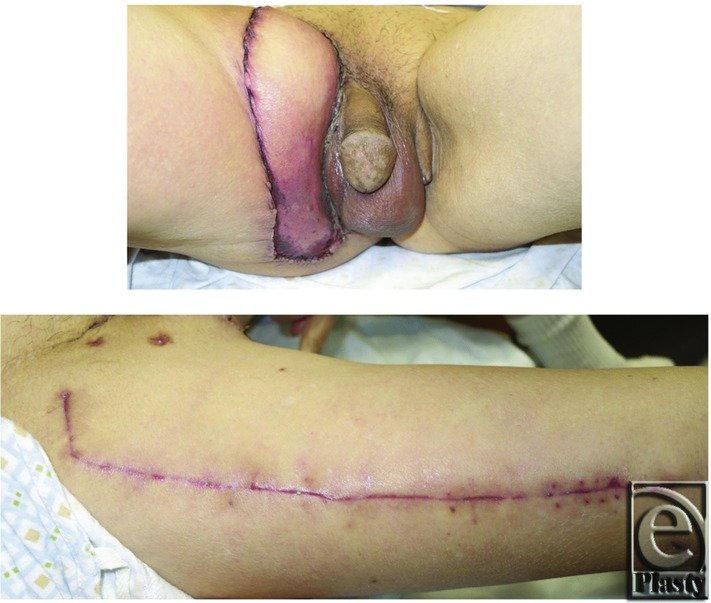
Healed ALT flap after surgery. (Top) ALT flap reconstruction of penoscrotal defect with good coverage, no contracture, and good functional result. (Bottom) Well-healed donor site with good thigh strength and no loss of sensation.

At postoperative follow-up, there was a small area of wound breakdown at the distal portion of the flap. There was no evidence of infection. Minimal local wound care was provided, resulting in a healed wound with good contour, no contractures, and full ability to ambulate without difficulty. At 6-month follow-up, the patient was able to resume all of his normal daily activities (Fig 2).

## DISCUSSION

Our current knowledge regarding the true incidence and prognosis of EMPD is lacking. Although the absolute incidence is difficult to determine due to insufficient data, one may speculate that the incidence of EMPD of the penis and the scrotum is high in Asians compared with other groups, as most of the larger studies are reports of Asian men.^7^^-^10 There is often a delay in diagnosis up to 3 to 4 years, as penoscrotal EMPD is often initially mistaken for contact dermatitis, psoriasis, fungal infection, seborrheic dermatitis, lichen sclerosis, melanoma, mycosis fungoides, and other skin lesions. There are reports of initial misdiagnosis in up to 92% of cases.^11^ Therefore, early skin biopsies are recommended to provide prompt treatment.^4^^,^^7^^,^^12^ Paget's cells are recognized on pathological assessment by a characteristic round, pale, vacuolated cytoplasm that stains strongly for mucin, with a large reticular nucleus located within the epidermis.^11^ It has been proposed that EMPD can be classified into 2 categories, with primary EMPD having no association with underlying neoplasm and secondary EMPD being associated with an underlying visceral malignancy.^13^^,^^14^ Previous studies have suggested that various immunohistochemical markers such as cytokeratin 7 (CK7), BRST-2, and CK20 may be useful in determining primary from secondary EMPD.^14^^,^^15^ However, a recent article by Perrotto et al^16^ indicates that the role of CK7, CK20, and BRST-2 is limited in discriminating the 2 groups, as they were all shown to be positive in large subsets of both groups, highlighting the role for clinical and pathological correlations.

There are multiple competing models of EMPD pathogenesis. One theory suggests that the Paget's cells arise from the epidermis and extend into the contiguous epithelium of hair follicles and eccrine sweat glands.^17^ An alternative theory suggests that the nidus is an adnexal carcinoma or internal malignancy that subsequently spreads into the contiguous epidermis.^18^^,^^19^ As EMPD and mammary Paget's disease have similar features, the initially proposed epidermal spread from an in situ or invasive malignancy in an adnexal gland seemed analogous to mammary Paget's disease arousing from ductal carcinoma in situ. However, unlike mammary Paget's disease, a much smaller proportion of cases of EMPD involve an underlying adnexal carcinoma.^8^^,^^20^^-^22 Another theory states that EMPD may have a multifocal origin or develop from intraepithelial metastasis, and this is supported by local recurrence after confirmed negative margins, with no evidence of internal malignancy.^6^ Another reported cause of EMPD is thought to be due to malignant transformation of intraepidermal cells as they differentiate into the apocrine phenotype. This hypothesis is consistent with the unique distribution of Paget's disease in the perineum, axilla, genitals, and breast areola.^3^ Despite the multitude of proposed theories, support for each can be found throughout the literature, and larger prospective studies with long-term follow-up will be useful for clarification.

The relationship of EMPD to adnexal carcinoma and internal malignancy is not clear. Underlying adnexal carcinoma varies depending on location, with a frequency of 14% to 20% in vulvar EMPD^20^^,^^21^ and 50% to 86% in perianal disease^22^ and 21% to 30% in penoscrotal disease.^6^^,^^8^ Although rare, underlying primary adenocarcinoma of a skin appendage is a predictor of poor survival.^4^ Chanda^13^ noted a 24% incidence (46 of 194 patients) of cutaneous adnexal carcinoma in those with EMPD and a mortality rate of 46% in those with underlying adnexal carcinoma compared with 18% in those without. The study cohort consisted largely of patients with vulvar and perianal EMPD, so conclusions about the mortality rate in those with penoscrotal EMPD were unclear. The author also investigated the relationship between the sites of EMPD and associated internal malignancy and found an 11% incidence of internal malignancy (prostate cancer and hypernephroma) in penoscrotal disease, 25% incidence in perianal disease, and 9% incidence in vulvar disease.^13^ Other reports have noted similar findings, with a frequency of concomitant internal malignancy of 15% to 50%. On the basis of these data, it was previously recommended that the clinician consider a directed internal malignancy search, which for penoscrotal disease would focus on the male genitourinary tract.^13^^,^^23^ A recent study by Zhu et al^4^ noted no evidence of urogenital cancer in 38 patients with penoscrotal EMPD, and other recent reports note an incidence well below 10% of associated internal malignancy with penoscrotal EMPD. Given these newer findings, the incidence of internal malignancy in penoscrotal EMPD appears to be much lower than previously reported and investigation for internal malignancy is not warranted unless there is involvement of the glans of the penis.^7^^,^^8^

Multiple treatment modalities have been used in isolation and in combination including WLE, Mohs micrographic surgery (MMS), external beam radiation, carbon dioxide laser, and chemotherapy to treat EMPD. Surgical excision is the standard treatment option for EMPD in all locations, but recurrence is common and has been reported up to 34% to 44% in some series.^24^ The Zhu et al^4^ analysis of 38 cases of penoscrotal EMPD treated with WLE with a 2-cm margin and FSA at ill-defined margins noted a 40% positive microscopic margin when only a conventional 2-cm clinical tumor-free border was used. Six of 38 patients (16%) experienced disease recurrence at 33-month follow-up.^4^

In the report by Hatta et al,^25^ they determined that the presence of nodules in the primary tumor, clinically detectable lymph nodes, elevated carcinoembryonic antigen levels, tumor invasion level, and lymph node metastases were significant prognostic factors in men and women with EMPD. The elevated carcinoembryonic antigen level and the level of tumor invasion were the only factors significantly associated with reduced survival, as the elevated carcinoembryonic antigen level was only seen in patients with systemic metastases. They saw no significant difference in the rate of local recurrence whether a wide surgical margin (>2 cm) or a narrow surgical margin (<2 cm) was used and no difference if intraoperative FSA was used. On the basis of their findings, they did not recommend an extensive surgical resection to reduce local recurrence; however, this study looked at all sites of EMPD, with no specific recommendations for penoscrotal EMPD.^25^

Focusing on penoscrotal EMPD, Yang et al^7^ reported that initial management with WLE with 1 to 2 cm of grossly uninvolved margin resulted in 17 of 23 cases (74%) with positive surgical margins and 9 of these 17 patients (52%) experienced local recurrence. They then began to use intraoperative FSA and noted 1 of 13 patients (7%) with a positive surgical margin and no local recurrences at 3-year follow-up. There was no local recurrence reported in patients with negative surgical margins. They found no correlation with depth of invasion.^7^ Kodama et al^26^ also reported a reduction in recurrence by up to half after surgical excision guided by the liberal use of intraoperative FSA. These studies highlight the importance of intraoperative FSA.

Lai et al^8^ examined 33 patients with penoscrotal EMPD treated with WLE and intraoperative FSA. They classified patients into 3 groups: (1) disease limited to epidermis; (2) involvement of an adnexal gland or hair follicle; and (3) presence of underlying adnexal carcinoma. There were no positive surgical margins. There was a 19% recurrence rate in those treated with WLE. There was no local recurrence or metastasis reported in those with EMPD localized only to the epidermis. The overall mortality rate in patients with underlying sweat gland carcinoma was 29% compared with 4% in those without. Although some early studies related only recurrence and prognosis to incomplete resection, they reported a statistically significant correlation between local recurrence and prognosis with the pathological depth of invasion and presence of underlying adnexal carcinoma, similar to that seen in newer reports.^8^^,^^13^^,^^27^

In 2008, Zhang et al^11^ noted no statistical difference in the rate of metastasis to groin lymph nodes between those with and without dermal invasion of penoscrotal EMPD. In the penoscrotal EMPD case series by Wang et al,^12^ all patients underwent a WLE with a 3-cm lateral margin. Eighty-one patients had long-term follow-up, and 8 patients (10%) experienced EMPD recurrence in less than 5 years. Recurrence rate was higher in those with a positive margin (56% vs 4%). The local recurrence rate of 10% is much lower than that seen in other studies with rates ranging from 16% to 44%.^7^^,^^8^^,^^24^^,^^28^^,^^29^ On the basis of the characteristics of EMPD having a multicentric origin and satellite lesions, they recommended using a 3-cm surgical margin and FSA for pathological examination in patients with unclear lesion borders.^12^ At this time, prophylactic lymphadenectomy or adjunctive lymphadenectomy is not recommended in patients with clinical negative groin lymph nodes. In the setting of clinically enlarged inguinal lymph nodes, a sentinel biopsy is recommended and a complete inguinal lymph node dissection should be performed in the presence of positive histology for metastasis.^6^^,^^11^

Radiation therapy has not provided clear results of cure based on prior studies.^30^^-^33 With regard to MMS, O’Connor et al^34^ reported local recurrence in 1 of 12 patients (8%) with MMS compared with 18 of 83 patients (22%) with WLE in a 24-month follow-up. While MMS does appear to compare favorably and provide good margin control when trying to preserve surrounding normal tissue, the longer operative time to allow for complete examination of large specimens may have a negative effect on patient morbidity and cost of treatment. In addition, personnel and facilities for the specialized technique may not be widely available.^34^ The noncontiguous nature of EMPD is a concern for recurrence seen in MMS. Topical treatment with imiquimod cream or 5-fluorouracil has also been suggested. Reports suggest symptomatic and clinical improvement, but margin control and clearance remain a concern.^8^^,^^11^^,^^35^ Laser therapy has also been described for penoscrotal EMPD, but its role in the treatment is not clear at this time.^36^^,^^37^ The alternative therapies to surgical excision have been used as primary treatment and may be quite useful in patients who are poor surgical candidates.^38^ However, at this time, surgical excision is recommended and other modalities are considered as adjuvant therapy based on clinical appropriateness.^7^^,^^8^

Following resection of penoscrotal EMPD, the defects can be rather large due to the delay in diagnosis precluding primary closure. There are many options for soft-tissue reconstruction of penoscrotal defects, and a simple and frequently used method is split-thickness skin grafting, which has yielded acceptable functional and aesthetic results.^39^ Advocates of skin grafting note concern for bulky soft tissue and skin color mismatch when flaps are used. The disadvantages of skin grafting a large area include decreased skin graft take secondary to contour, scar contracture, and potentially painful erections. In 1989, Lai et al^40^ used the iliac flap based on the superficial circumflex iliac artery to cover penoscrotal wounds ranging from 77 to 126 cm^2^, reporting advantages of a thin large soft-tissue flap that could preclude scar contracture. Qin et al^5^ discuss the technique of scrotal skin flaps for reconstruction of penoscrotal EMPD defects ranging from 48 to 96 cm^2^. In a small series of penoscrotal EMPD, Chen et al^6^ utilized the ALT flap. They utilized this flap in 5 cases where the wound was large and deep. In this situation, split-thickness skin graft would have provided poor coverage. Their patients experienced no severe complications with the ALT flap, only noting that 1 of the 5 patients complained of paroxysmal neuralgia and skin numbness of the lower limb postoperatively.^6^ The anatomy of the flap and harvest techniques were originally described by Song et al^41^ and have been further developed in subsequent studies.^42^ The ALT flap has been used in the reconstruction of multiple soft-tissue defects in the head, neck, extremities, and perineum.^42^^,^^43^ To our knowledge, the Chen et al^6^ series is the only report of the ALT flap being used to reconstruct penoscrotal defects after EMPD excision. For our patient, given the large wound (240 cm^2^) and contour defect, we elected to proceed with the ALT flap. The advantages of a large hair-bearing skin paddle, long vascular pedicle, acceptable minimal donor site morbidity, bulk associated with subcutaneous fat and skin, and short operating time made the ALT a most reasonable choice for our patient. Given the high incidence of recurrence, the ALT flap can be reelevated for reexcision of involved tissue whereas a skin graft cannot be reelevated. These factors all weighed heavily in our decision making.

## CONCLUSION

Penoscrotal EMPD is mainly observed in the elderly and may be more frequently observed in those of Asian descent. It is a rare disease with an indolent course that is often initially misdiagnosed. On the basis of review of the literature, the prognosis is good when there is noninvasive disease; however, invasive disease may indicate a more advanced process with associated poor prognosis. More detailed prospective studies are needed to further elucidate the impact of prognostic factors such as depth of invasion. There are several options for treatment; however, WLE and immediate reconstruction appear to be the preferred treatment option at this time for penoscrotal EMPD. A positive margin appears to be a risk factor for local recurrence, and FSA is often recommended to help achieve pathologically clear borders during excision. Each current reconstruction technique has its own associated risks, benefits, and complications. While split-thickness skin grafting is the most common method of reconstruction, it may not be the best method for all defects. It is key that the surgeon carefully assesses the wound in its entirety includxing involved structures, depth, and size following resection. The goal for reconstruction is to restore form and function, and while the ALT flap is not widely utilized, it may be a more useful tool in the armamentarium of caring for patients with penoscrotal EMPD than previously considered.
